# Progress in Ophthalmology

**Published:** 1900-01-27

**Authors:** 


### PROGRESS IN OPHTHALMOLOGY.

(*Continued from page 260.*)

**Eye Symptoms in So-called "Hay Fever."**—John Hern[4] at the same meeting read a paper with the above title, and at the beginning said: As a rule, only neurotics have the trouble. About the second week in June, following a ride or drive in the country, suddenly there arises an intense itching and burning sensation at the inner canthus, usually bilateral. The patient feels impelled to rub or scratch the eye, which "feels as if filled with heated sand." The whole conjunctiva is seen to be swollen and œdematous, especially at and around the inner canthus, and if the swelling extends quite up to the corneal edge there usually is considerable photophobia. The œdema lasts about two or three hours, and then gradually subsides, provided the eyes are rested absolutely. At night the patient can usually sleep for two or three hours, and then awakes with pains and the "sand feeling" in the eyes again. Application of 2 per cent. cocain gives relief sufficient to enable the patient t6 go to sleep again, but only to be reawakened in two or three hours. Unless sent to the seaside or on a voyage these symptoms continue for six or seven weeks and then disappear, irrespective apparently of any treatment, but only to reappear next season. Treatment is difficult and unsatisfactory as far as medicaments of any kind are concerned. A few hours at the seaside relieves some. A month's voyage seldom fails to cure for that season. Boric cream 2 to 4 per cent. gives relief, but relief only. A solution of boric acid with spt. of nitrous ether ʒij. to ℥iv. also relieves. If the lids are granular this should be treated in the intervals, as it always aggravates the symptoms. Mr. Devereux Marshall said he had had considerable experience of the symptoms from his own personal experience. He had found that its development was most irregular, and all treatment very ineffective. He severely condemned the use of cocain, as he found that people who used it for the eyes and nose invariably required to increase the strength, and as they swallowed and absorbed a great deal of cocain they soon began to feel the general effects of the drug, and, consequently, they laid the foundation of the cocain habit, which he believed to be far more widespread and harmful than was generally supposed.

**Sudden Paralysis of the Third Pair of Nerves and Interstitial Keratitis in Acquired Syphilis.**—Fromaget[5] reports an interesting case of ocular complications of syphilis in a woman of 39. She had been suffering with recent interstitial keratitis, and while under treatment was suddenly affected with complete paralysis of the third pair of nerves. Going to bed apparently all right, with the exception of keratitis, she awoke next morning with ptosis, external strabismus, dilated and inactive pupil, in fact, involvement of the entire external and internal muscles supplied by the third pair. It seemed as though not a single fasciculas of the nerves had been spared. Under full doses of the iodides, electricity, and intramuscular mercurial injections, there has been a gradual improvement in the paralysis, but by no means a recovery. The corneal condition had improved markedly. Fromaget believed that, on account of the rapid onset and complete involvement, the paralysis was due to hæmorrhage of the nerve trunks. He thinks had the paralysis been less extensive a nuclear lesion might have been suspected, but certainly the rapid onset precludes the possibility of neuritis exostosis, gumma, or other intracranial syphilitic lesion being the cause.

**Atrophy of the Optic Nerve following Profuse Hæmorrhage.**—Theobald[6] reports the case of a man 57 years of age, who had present for 20 years symptoms of gastric derangement, with frequently repeated attacks of vomiting. Following copious hæmorrhage from the stomach, repeated twice at intervals of 48 hours, the vision, which had previously been good, became greatly impaired. Subsequently there was some improvement in sight, but later on again it grew worse. The patient admitted having been a pretty constant smoker, but denied drink or syphilis. On exploration, a nodular mass was found near and partly occluding the pyloric orifice, which was regarded as a malignant growth developed in the cicatrix left by a former ulceration. On ophthalmoscopic examination, both optic nerves were found in a state of advanced atrophy, with some cupping, and marked contraction of the retinal arteries. The optic discs had a woolley appearance, their outlines were irregular and ill-defined, and in each eye there were pigment patches in the retina, not only about the margins of the discs, but at points some distance from it, and especially in the macular region, indicating that the atrophy of the nerve had been preceded by an inflammatory process which had involved the retina as well. From a study of this case and the literature to hand on the subject, the following conclusions were drawn:—

1. That the weight of evidence afforded by the ophthalmoscope points to thrombosis of the central retinal artery as the usual cause of the blindness that attends post-hæmorrhagic anæmia.

2. That the resistance offered to the already enfeebled blood current in the central retinal artery by the intraocular tension is an important etiological factor in determining this result.

3. That in exceptional cases the ophthalmoscope indicates that the thrombosis occurs, not in the artery, but in the central retinal vein,

4. That in other exceptional instances it may be that the loss of sight and the ophthalmoscopic appearances that accompany it are the result of a hæmorrhagic or serous effusion into the optic nerve or its sheath; and here again the obstruction or damming back of the blood-current in the retinal artery by the intraocular tension probably have much to do with bringing about this result. In the case reported the question naturally arises whether the long-continued gastric disturbance and the existence of malignant disease may not have been at least a predisposing factor in the development of inflammatory and degenerative changes in retina and optic nerve.

[4] Brit. Med. Jour., Sept. 23, 1899. [5] Inter. Med. Jour., July, 1899.
[6] Med. Rec., Sept. 9, 1899.

